# In the interest of food safety: a qualitative study investigating communication and trust between food regulators and food industry in the UK, Australia and New Zealand

**DOI:** 10.1186/s12889-017-4118-x

**Published:** 2017-02-13

**Authors:** Samantha B Meyer, Annabelle M Wilson, Michael Calnan, Julie Henderson, John Coveney, Dean McCullum, Alex R Pearce, Paul Ward, Trevor Webb

**Affiliations:** 1 School of Public Health and Health Systems, University of Waterloo, Waterloo, N2L3G1 Canada; 2 Discipline of Public Health, Flinders University, Adelaide, Australia; 3 School of Social Policy, Sociology and Social Research, University of Kent, Canterbury, UK; 4 School of Nursing and Midwifery, Flinders University, Adelaide, Australia; 5 Food Safety and Nutrition Branch, SA Health, Adelaide, Australia; 6 Department of Knowledge Integration, University of Waterloo, Waterloo, Canada; 7 Food, Information, Science & Technology Branch, Food Standards Australia New Zealand, Canberra, Australia

**Keywords:** Food industry, Food regulation, Food safety, Trust

## Abstract

**Background:**

Food regulatory bodies play an important role in public health, and in reducing the costs of food borne illness that are absorbed by both industry and government. Regulation in the food industry involves a relationship between regulators and members of the industry, and it is imperative that these relationships are built on trust. Research has shown in a variety of contexts that businesses find the most success when there are high levels of trust between them and their key stakeholders. An evidence-based understanding of the barriers to communication and trust is imperative if we are to put forward recommendations for facilitating the (re)building of trusting and communicative relationships.

**Methods:**

We present data from 72 interviews with regulators and industry representatives regarding their trust in and communication with one another. Interviews were conducted in the UK, New Zealand, and Australia in 2013.

**Results:**

Data identify a variety of factors that shape the dynamic and complex relationships between regulators and industry, as well as barriers to communication and trust between the two parties. Novel in our approach is our emphasis on identifying solutions to these barriers from the voices of industry and regulators.

**Conclusions:**

We provide recommendations (e.g., development of industry advisory boards) to facilitate the (re)building of trusting and communicative relationships between the two parties.

## Background

Food regulatory bodies play an important role in the field of public health, and contribute significantly to the health of populations in a variety of ways [1]. In particular, their role involves both developing food safety standards, and/or enforcing these standards to monitor food safety risks. There are two main types of food risks for consumers. The first relates to hygiene and is specific to foods being unsafe for consumers to consume. The second relates to food standards which includes nutrition labelling (e.g., consumers are misled over fat content), safety labelling (e.g., use by dates are incorrect), and composition labelling (e.g., labelling misleads the consumer because contents do not meet compositional standards) [2]. As such, food safety risks refer to those that directly affect human health, but also risks regarded as “food fraud incidents”, relating to deception or malintent [3]. As a result, the role of food regulators is broad and can range from enforcing food standards in restaurants to organising large scale food recalls. Regardless of role, food regulation is essential for ensuring public safety as well as developing and maintaining public trust in food [4].

Enforcement involves engagement with members of the food industry who are required to comply with such standards, with penalties occurring if noncompliance is identified. Food safety incidents, for example Garibaldi (Australia), bovine spongiform encephalopathy (BSE) (UK) and toxic levels of iodine (New Zealand) [5–7] have brought to light the importance of regulation of the food industry [8, 9]. The costs associated with foodborne illness are important for both regulators and industry. Using Australia as an example, an estimated 4.1 million domestically acquired cases of foodborne gastroenteritis occur annually, costing an estimated $1.2 billion AUD per year [10]. These costs are absorbed by a variety of actors with a stake in food safety, including businesses (food safety recall costs total $14 million AUD) and the Australian government ($10 million AUD) [11]. As such, both regulators and members of industry have a stake in maintaining food safety standards and avoiding food scares.

Regulation in the food industry involves a relationship between regulators and members of the industry, and it is imperative that these relationships are built on trust. At its core, trust is described by social theorists as existing in three parts: A trusts B to do X [12, 13]. In this context, A and B are the regulators and members of industry, and “doing X” (either A for B, or B for A) can be described as acting in each other’s best interests. No matter the industry, trust has been shown to play a very important role in regulation-industry relationships, enhancing cooperation and leading to better outcomes for both parties [14]. Research has shown in a variety of contexts that businesses find the most success when there are high levels of trust between them and their key stakeholders [15]. Indeed, it is in the best interest that the two parties cooperate. Industry’s trust in regulation, and subsequent compliance with food safety standards may minimize food safety incidents that have the potential to reduce consumer trust, reducing their profits. For regulators, having a trusting relationship with industry may lead to greater transparency in times of food scares or when industry are finding it challenging to meet food safety standards.

Trust is a complex multidimensional concept consisting of both a rational component (arising from experience) and an irrational component based on instinct and emotion [16, 17]. Importantly, trust can be understood to occur at two distinct levels – institutional [18] and interpersonal [19]. Institutional trust is that which is placed in one or more social systems or institutions (e.g., Food Standards Australia New Zealand). Interpersonal trust is negotiated between individuals; for example, between a consumer and a grocer. Both forms of trust are important for understanding where and how trust can be (re)developed and maintained in the context of food safety regulation and compliance. Both the reputation and knowledge of the institution (e.g., Food Standards Agency or McDonalds), as well as the personal relationships with those who represent it (Food Safety Manager or Restaurant Owner), are vital to the pursuit of trust between members of industry and regulators.

The relationship between regulators and industry can be complicated by a variety of factors, including the varying sizes of businesses involved, as they can range from multinational corporations to small, family-owned businesses [20]. Therefore, it is important to recognise both the perceived and real barriers that the food industry face when aiming to comply with food safety standards, and the level of understanding of these challenges from the perspective of regulators. An evidence-based understanding of the barriers to communication and trust is imperative if we are to put forward recommendations for facilitating the (re)building of trusting and communicative relationships with the aim of increasing compliance in the interest of public health.

This paper presents the views of regulators and members of industry regarding their trust in and communication with one another within three countries (UK, New Zealand, and Australia), which necessities a brief overview of the policy and institutional context within each locale. Food Standards Australia New Zealand (FSANZ) is a bi-national body that is responsible for the Australia New Zealand Food Standards Code which forms the basis of much of the food law in each country. FSANZ is an independent science-based organization that is managed by a Board whose members are experts in various aspects of food (e.g., toxicology, nutrition, microbiology, food technology, food industry etc.). FSANZ is not responsible for the enforcement of the Code, nor is it responsible for the policy that informs the direction of the Code – this is undertaken by the states and territories of Australia, and the Government of New Zealand. In the case of policy this is generally the departments of health and/or agriculture, while enforcement may be undertaken by local authorities. In the UK, the Food Standards Agency is generally responsible for the development of food safety policy and controls, while enforcement of these are devolved to local authorities. In England the nutrition components of food regulation are administered through the Department of Health.

For the purposes of this paper, the use of the term regulator is inclusive of both individuals responsible for developing food safety standards and for enforcing food safety standards. The term industry is inclusive of a variety of individuals and organisation types (e.g., grocers, small business owners, and large corporations). Our analysis of these perspectives has allowed us to identify barriers and facilitators to communication and trust, and potential areas of conflict that are problematic from a public health perspective that may be amenable to change. A variety of data-driven solutions are provided for (re)building trusting and communicative relationships between the two parties, with the end goal of protecting the public.

## Methods

### Study

The data presented in this in this paper comes from a larger study examining trust in the food system from the perspectives of food regulators, the food industry and the media, conducted across three countries – UK, New Zealand (NZ) and Australia (AU). A protocol paper outlining this wider study has been published elsewhere [20].

### Recruitment

Individuals working in the food regulation and food industry were recruited for this research. Recruitment was through purposive sampling, which enabled participation of individuals who were information rich [21] and had relevant experiences to share [22]. An initial list of people to contact was developed by the research team, based on their own contacts and knowledge of food industry and regulatory settings. Considering the varied roles of representatives working in the food regulation settings, a sampling strategy was developed to ensure coverage of participants working in different areas including policy development, standards setting, implementation, inspection and enforcement. Likewise, the sampling strategy for industry was developed to ensure coverage of a range of business types including local food industry, franchise food industry, supermarkets, industry advocacy groups and consumer advocacy groups. Potential participants and organisations were contacted by one researcher in AU and NZ and two researchers in the UK. Initial contact was made through e-mail. If no response was received, a second email was sent and this was followed up by a phone call if a response to the second email was not received. In total, 80 individuals were contacted. Eight declined participation or did not respond to requests for participation. The email/ phone call outlined the purpose of the study and invited the individual to participate in an interview. An information sheet and letter of introduction was also included. Table 1 provides an overview of the participant sample.

**Table 1:** Participant sample

UniteUKd Kingdom	New Zealand	Australia
Industry: *N* = 14	Industry: *N* = 4	Industry: *N* = 11
Regulator: *N* = 11	Regulator: *N* = 6	Regulator: *N* = 26

As noted above, the actors within the food industry, as well as regulation vary, which is an important consideration in the interpretation of the results. Table 2 provides an overview of participant characteristics as they relate to the participant’s role within regulation or industry.

**Table 2:** Sample characteristics^a^

Sector	Description of role	Examples of roles	Participant IDs
Industry	Individuals working at various role in the restaurant industry	e.g., food and beverage managers, chefs	AU-IND: 9-10
	Individuals working with industry in the role of food regulation, or in the role of consumer advocate, nutrition and food safety	e.g., consultants in nutrition, food safety, and regulation	UK-IND: 3, 5, 6, 7, 11, 13
			NZ-IND: 1
			AU-IND 1, 11
	Individuals involved in the finance and management of large scale corporations	e.g., director or CEO of food and grocery councils and multi-million dollar corporations	UK-IND: 1, 2, 4, 8-10, 12
			NZ-IND: 2, 3, 4
			AU-INDI: 2, 3, 5, 6-8
	Individuals involved in the production or manufacturing of food	e.g., director of quality assurance	UK-IND: 14
			AU-IND 4
Regulator	Individuals of varying levels of seniority from national food regulatory bodies	Food regulatory bodies	UK-REG: 1, 2, 7-8, 11
			NZ-REG: 5
			AU-REG: 3-7, 11-12, 14, 18, 25, 26
	State-based food regulators	State Health Departments	UK-REG: 3-6, 9, 10
			NZ-REG: 1-4, 6
			AU-REG: 1, 2, 8-10, 13, 15-17, 19-24

^a^In order to maintain participant confidentiality, we have not provided the specific names of the industry/regulator body within which participants are affiliated. Rather, we have categorized participants according to their role and job title for the purpose of data interpretation

### Data collection

Data were collected using semi-structured interviews. The interview guide was piloted separately in the UK, NZ and AU to check for usability and relevance to the cultural context. Minor changes were made to increase flow of the interview schedule based on feedback from piloting. The interview schedule was used as a guide for discussion during interviews. Relevant to this paper, the interview guide was designed to discuss food regulator and food industry representatives’ ideas about food safety, responses to food incidents in general, regulation and maintaining safety standards, as well as challenges to compliance with food regulation. The interview guide has been published elsewhere [23].

Interviews were conducted face-to-face or over the telephone at a time and location convenient for the participant. Phone interviews were used when participants were geographically distant from the interviewers. Interviews ranged from 30-60 min. Three interviewers collected data, one in Australia and NZ and two in the UK. The three interviewers met fortnightly via Skype during the data collection period to ensure consistency in questioning. Interviews were conducted in AU and the UK between July and November 2013 and in NZ in October 2013 until saturation of themes was reached [24]. Interviews were digitally recorded using a voice recorder after consent to conduct the interview had been obtained.

### Data analysis

Digital voice files were de-identified and transcribed verbatim. In this study, nonverbal cues, emphasis and pace were deemed less important and were not transcribed. Interview transcripts were checked by each interviewer for accuracy. De-identified transcripts were then imported into NVivo 10.0 (QSR International, Doncaster). A start list of codes was developed by the research team (including academics and industry partners working in the food regulation setting). Transcripts were then coded by one researcher using this start list of codes following six stages of thematic analysis [25]. Central to this paper are the codes developed from the research objectives that included role of interviewees in regulation, industry and maintaining safety standards. As coding progressed, further themes and sub-themes were added based on the objectives of the research and information in the data. Coding was checked and agreed upon by team members at fortnightly team meetings and at two data analysis workshops during the data analysis phase of the research. Other members of the research team reviewed up to five transcripts each to confirm the themes arising from the primary researcher’s analysis.

## Results^1^

### Regulators

The two themes identified in interviews with regulators related to their role in engagement with the food industry, and conflict and understanding in their relationships with industry. Not surprisingly, the regulator voices across all three countries represented below are from what we have categorized as ‘state-based food regulation’ (see Table 2), while those in the national food regulatory bodies did not comment on their engagement with the food industry. This is likely because it is the state-based regulators who are responsible for the enforcement of the code and therefore have ongoing personal engagement with members of industry.

### Perception of role in engagement with food industry

Participants across all three countries discussed their role in food safety, and their relationship with industry. There were clear differences in how AU and NZ described their roles when compared to UK regulators.

AU and NZ regulators identified their role as developers or enforcers of regulation, with the primary focus being public safety. However, they identified conflict with industry, which they perceived to be a misconception on the part of industry that regulators try to cause problems for industry:

I mean I think we’re all after the same end, like we’re all there to protect public health, and I think a lot of the food guys are wanting to do that as well…And, yeah, we don’t want to be shutting people down, we really don’t. If we can identify problems we want to go in and fix them up and I think if that can all be managed well and the people where a problem might be identified, if they’re very keen to do the right thing then often they’re the success stories and they actually – you know, their businesses go on to be stronger and stronger and stronger… There’s a perception perhaps that public health want to close down and anywhere that’s dirty we don’t want them to operate anymore and I don’t think that’s the case. (AU-REG23)

The above comment also identifies recognition of the mutual benefit that can result from compliance with regulation; public safety and making industry stronger. NZ-REG1 echoes this, commenting that while regulators are at times perceived as being the enemy, industry often do value their recommendations and view them as strengthening industry. In recognition of the potential to be viewed as the enemy, the following suggests that NZ regulators make a concerted effort to ensure their behaviours and actions suggest otherwise, and to work with industry to resolve issues:

Whenever we do – in government we tend to be fairly cautious because we’re bureaucrats and sometimes – there’s always a little bit of pushback [from industry]; sometimes there’s quite a bit of pushback. I have to say quite often companies are on the same page when they know that their reputation is at risk so quite often the companies – well, there was one of the companies that we were dealing with in the incident who said – we said ‘this is what we think is affected and this is what we recommend you recall’ and they said ‘we’re not going to quibble, we’re just going to get rid of the stuff. Anything that’s potentially affected we’re just going to withdraw the whole lot’ and the only reason they did that is for commercial reasons, not for trust reasons and I think that was the right thing to do. It will have cost them more money but it was an investment in the brand. (NZ-REG1)

UK regulators also identified their role as being responsible for enforcing regulation. However, within the following quote there is no indication that working with industry may be beneficial for both parties. The quote, among others, is indicative of a more top down approach than identified in NZ and AU interviews:

I think there’s an expectation on their part [industry] that we’re almost viewed as a consultant in many cases and we should be providing them with information and guidance to update their systems when in fact that responsibility lies with them and they’re duty bound, or they need to think about who they engage as a consultant to assist them in their activities. It’s not our role really; our role is to identify what’s not compliant and to advise the businesses that they need to address these issues. You might offer some guidance but our role is not to recreate or reproduce your documentation or your procedures or practices, it’s to make sure that they are correct and advise you of that and then monitor the activity to see that you’ve addressed those issues (UK-REG5)

UK regulator interviews demonstrated a clear division of roles regarding food safety, and little communication or partnership between industry and regulation.

### Perceived influences on regulator-industry communication: conflict and understanding

Within the UK specifically, regulator interviewees discussed the reluctance of industry to work with regulators. For example:

They [industry] have the technical knowledge and we may not have the knowledge to deal with some of the stuff because it is quite technical… we have a general understanding of most stuff and if we want to obtain particular information we have to go through loads of regulation and guidance to get that information where they’re actually doing it all the time. Sometimes you may walk in and they’ll say ‘well, what do you know? I know more about this activity than you do’ which they may do but we will look at it objectively and where our powers or where our skill comes in is an ability to audit systems and to look at stuff objectively and make decisions based on the information that’s provided to us. (UK-REG5)

In relation to the recent horsemeat scandal in the UK (2013), the following was stated regarding why communication might lead to conflict between the two parties. A clear explanation is given which relates to the fear of repercussions that may result from transparency between the two.

I think that at the start there was quite a bit of reluctance of industry to work with the < name of regulating body > and that’s sort of quite historic because they were concerned that if they told < name of regulating body > things and they admitted they didn’t do things right they might get punished. (UK-REG10)

UK regulators identified industry as falling into one of four groups; dependent on the extent to which they *want* to comply with regulation, and the extent to which they actually *do* comply with regulation. The perception is that these different ‘types’ of industry require differential treatment:

You’ve got those that will know what they need to do and how they need to do it and will do it proactively; you’ve got those that want to comply but don’t really know how to comply and are looking to you for help and advice; you’ve got businesses that don’t want to comply but, you know, with a little bit of sort of help the persuasion will get there. And you’ve got those that don’t care, don’t want to care, are in it for pure profit and are trying to avoid the regulation and trying to avoid being caught making money at the expense of, well, anything really. (UK-REG3)

Those who are trusted to comply were identified as requiring less monitoring:

So the likes of Tesco and Sainsbury’s and McDonald’s, etcetera, you would probably be able to look away from them because they have got generally very good systems in place when they’re implemented…leave the likes of the bigger retailers to their own devices because simply we knew that they would do their own investigation and pull this stuff if they found it on their shelves. (UK-REG3)

However, conflicting with the above quote, the following suggest that larger industry (e.g., McDonalds) are more closely monitored because of the greater impact if a food incident was to occur. UK-REG3 continues:

… if you have somewhere that has the potential to have a bigger impact you visit it more often than you would somebody that has the potential of a smaller impact, like a post office selling a few lollies; that makes common sense. (UK-REG3)

We are unable to explain this inconsistency and further understanding of the flexibility in monitoring and enforcement is required.

Whilst also identifying the conflict between regulators and industry, AU regulators were sympathetic to the fact that compliance with (over)regulation can be burdensome and potentially detrimental to business. The following quote emphasizes the influence that the political economy has on industry food safety management:

A lot of people don’t want to make – no-one really wants to make somebody else sick but there are business imperatives that are pushing some practices, you know, they need to make money to survive because that’s their livelihood but also they need to be doing those things in a safe way that doesn’t make people sick. (AU-REG23)

### Industry

#### Difficulties in compliance

Representatives of industry discussed their difficulties with compliance in relation to the changing nature of regulations, the knowledge gap between industry and regulators, a lack of consistency across regulators, and concerns about the potential for over-regulation. Not surprisingly, the majority of the comments regarding difficulties in compliance were from individuals whose primary position in industry was the finance and management of large-scale corporations (see Table 2).

NZ and AU industry interviewees discussed the difficulty in abiding by complex and sometimes irrelevant regulations that are constantly updated and changed, requiring more work on the part of the industry to get up-to-speed.

We need to be making sure that we produce a product that is safe and we don’t need to complicate that. Especially for small producers let’s just keep it simple. Let’s just make sure that all the criteria is being met but let’s not overcomplicate it. (AU-IND4)

I think they’re updating the current food standards codes. I think it’s been challenged that many times that it’s irrelevant to the industry…Because it’s boring and you have to read ten pages to get to the results. (AU-IND5)

Contrary to regulator beliefs about their proximity to and understanding of industry challenges, industry participants noted that regulators are too far removed from the food industry and therefore, do not relate to the barriers to compliance with regulation:

Well, they need to understand food and they can’t understand food if they don’t understand the food system that’s providing it, particularly if you’re trying to protect consumers, which is their fundamental role, of course. (AU-IND3)

Consistent with comments made by AU industry, UK interviewees also commented that individuals in regulation do not always have the food expertise required to make the decisions they are faced with:

I think it’s very important to have an independent position but you need to work closely because if you want real expertise and technical guidance, technical advice, some of that best knowledge sits within the food chain. You have to find a balance between the two but independent robustness is very important. (UK-IND9)

They get in wrong but in terms, sometimes, of their understanding of the industry. You know, a lot of their expertise has been lost down the years as people have moved around, gone, and they sometimes seem to know remarkably little about how the food supply chains work. (UK-IND13)

This lack of knowledge on the part of regulators was seen as problematic and in some cases, harmful to businesses. For example:

I mean the regulators, sometimes they’re not always as sensitive to the sort of brand issues as food manufacturers are and they can say something which is intended for the best but, you know, plays rather badly in the media and can sometimes make matters worse. Sometimes I’ve had incidences where they have made statements in the media which have required retailers to remove product from shelf completely unnecessarily because they’ve just said the wrong thing completely inadvertently because they’re not trained to deal with the media or they don’t sort of think through what it is they’re actually saying. (UK-IND12)

Another difficulty faced in complying noted by industry was the lack of what they referred to as ‘consistency’ in enforcement, which in some circumstances suggested a conflict of interest or differential treatment:

Environmental Health Officers in different council areas may be administering the law differently – they need to come together and apply the law in the same way in order to get a unified approach. (AU-IND2)

I think also enforcing [regulation] it is quite important as well. I think one of the things that people get upset with – you know, again I refer to my friend who manages a pub – she says ‘oh well I’ve been into restaurants and I’ve walked past their kitchens and I’ve seen their kind of messy floors and how come I’m being pulled up because I didn’t fill in my fridge/freezer temperature gauge for last week and yet they had meat on the floor? When I walked past I could’ – so I think there’s that. (UK-IND4)

Concerns about the extent of regulation, and the potential effects of regulation on smaller industry were also noted:

Also the cost of manufacture in Australia is ridiculous with all the red tape. All the requirements that the government puts in, day in day out, it doesn’t help local manufacturers. If you’re small it’s really hard because you’ve got no volume. It’s easier for bigger players because they can bend the rules in different ways because they’ve got volume. (AU-IND5)

I think farmers are very fed up with red tape and bureaucracy. We don’t want to tie people down with lots of bureaucracy. It makes it harder to run a business with all that, when you’ve got paperwork and bureaucracy so I think – I don’t think it’s an issue of trust, it’s just an issue of focus. So farmers want their focus to be on driving these businesses forward not holding them down by red tape and bureaucracy… regulators in the UK are very – can definitely put a lot of burden on farmers and that’s not always equal to other parts of the world which makes it difficult for us to compete globally. (UK-IND10)

While not a matter of distrust, the above identifies conflict or a lack of communication between the industry and regulators, and the perception or reality that regulation is problematic for business.

### Conflict with, and distrust in, regulators

Several participants from NZ and AU industry noted the importance of trust in their relationship with regulators, but indicated that trust between the two is not always present. Among industry representatives, this was largely attributed to poor communication and engagement by the regulators, which in turn led to the perception that regulators are not focused on maintaining public safety, as intended:

I’m not a big fan of food regulators. I think food regulators are there to protect corporate industrialized food systems and not public health and safety. You don’t have to look very long at the regulatory system or at the risks that we are exposed to to start to question the regulatory system and to realize that the regulatory system is letting us down, letting the public down and then the consequence of that is you have low trust in mainstream – a mainstream food system and the regulatory authorities that oversee that food safety regulatory systems to be overhauled, completely overhauled to work on the premise of protecting public health and safety rather than, as we do at the moment, protecting industry…. I mean chemicals used in agriculture, in food production, chemicals used in food processing, GMOs used in foods, radiation being used, these are all issues which we believe are being very poorly assessed. System wide risks that the public is being exposed to that’s being very poorly assessed for public health and safety because the regulatory system is designed to not look at what they don’t want to look at. It’s designed to hide under the carpet many of the consequences of the risk that the food industry is using or technologies that expose the public to risks. Many of those risks, the regulatory system desire to not look at those risks. (AU-IND1)

The interviewee here, whose role in industry is food safety, is not surprisingly focused on ensuring public safety. Their perception is that regulators focus too much on protecting selected industry, suggesting that Australian food regulation is in part at least, driven by the political agenda. Distinct from the role described by regulators, their perception is that the safety of the public is not the primary concern. Similarly, NZ-IND4 discusses their distrust in regulators, but with a different rationalization which may be related to their role in the finance and management of industry. The quote suggests that trusting regulators to take action can have negative implications for industry when the safety of a product is in question:

It’s not just what the company does, it’s also what the regulator does and if I look at the < removed for confidentiality > recall I believe that brand has sustained some damage through no fault of the company at all, purely because < name of regulatory body > pressured into recalling every single batch, its entire brand line, when in fact there were only specific batches involved; that said to consumers there’s something wrong with every product. (NZ-IND4)

UK-IND11, identified as a consumer advocate, also discussed the fallibility of regulators and the consequences of these alleged mistakes on industry. The participant is sympathetic to the dual role of regulators as looking out for consumers but also having to consider the implications for industry if their actions are overly cautious:

The problem I think with regulators is in the Catch 22 where they can take proactive action but if that means that they, for example, go too early and say to companies ‘okay, clear the shelves. Those tens of millions of products, huge amounts of your money, off the shelves, chuck them away, destroy them; it’s your profit’ and then it turns out that it wasn’t really a problem and the regulator had – you know because there’s always uncertainty about these things, the regulator had kind of erred on the side of caution, can cause, you know all kinds of problems and so regulators aren’t allowed to do that because they have to sort of pre-empt things but also know for certain. (UK-IND11)

The dual role of the regulator, as advisor to the consumer and industry, was also identified as a potential source of conflict with regulators. For example, the case above identifies regulators as being perhaps overly cautious for the protection of the consumer, thereby damaging the industry. Below, NZ-IND1 who is involved in the finance and management of industry, identifies the pressure on regulators to serve multiple interests:

I mean food regulators, they can find themselves trying to serve both consumers and manufacturers. We have quite a strong push here for export so a lot of focus on the safety of food exports, so that can mean that the domestic market is – domestic consumers are less well served. (NZ-IND1)

NZ-IND1’s comment might also relate to concerns posed above regarding regulators’ focus on the political agenda, rather than public health. If there is a push to export food and to grow business for economic gain in NZ, there is a potential for conflict of interest – however, this is not empirically supported and is in need of further exploration.

UK industry also noted the importance of an interpersonal relationship between industry and regulators, and the importance of trust. Trust was however identified as having declined over time:

I think it’s [trust] not as good as it was. I mean in days gone by the links were a lot closer with the Minister of Agriculture, as it was then, but a lot of it’s down to personal contacts and that’s why it’s important for companies to have people who build those contacts, that you pick up the phone to people and there’s a level of trust there. (UK-IND7)

This point was further emphasized by UK-IND3 who noted that as the result of a change in government, individuals in food regulation no longer communicate with the food industry:

They had a food policy which was a much more – I mean they never got to their conclusions but at least they started the process of a much more comprehensive food strategy really. This government abandoned all that and went back to, you know, ‘how do we produce more and sell more in Britain?’… the previous government used to hold six monthly discussions with CEOs of food retailers; this government abandoned that straightaway and all they wanted to talk to was farmers all the time, which was fine because actually it means they bother us less, until of course you get an incident and then they’ve got no knowledge of how our sector works or our relationship with consumers and what actually happens on labels and all these kinds of things. (UK-IND3)

The lack of communication with the food sector may be in part due to the means by which the role of regulators is governed. Consistent with comments from AU industry about the role of government in shaping the agenda for the food sector, UK-IND3 (industry food regulator) and UK-IND2 (finance and management) comment:

Well I would say our relationship with the officials is very good… but the ministers are not necessarily focused on our end of the supply chain. They’re very politically driven and they’re politically driven towards farming rather than either manufacturing or retail… (UK-IND3)

I think certainly government and UK government and some of the key government departments we’ve got individual good relationships there but in terms of how much they listen and respond to not just us but lots of other organisations in the food and farming space, so that’s open for debate I think. Certainly we would say it’s difficult to engage UK government on lots of these issues. (UK-IND2)

Here it is presented that the interests of the government, in this case selling more product within Britain, needs to be taken into consideration. UK-IND11’s comment emphasises the power of political agendas:

But also you know, there’s huge legal things and political things because then you know if the politicians lose the trust in the regulators you know they’ll end up restructuring the organisation......So there is a thing where regulators I think are in a very, very difficult position…(UK-IND11)

UK industry viewed the extent of bureaucracy and the fragmented information spread amongst stakeholders as problematic to the functioning of regulation:

You know, regulators and government, it’s so difficult to do that and there is that thing of always, you know, kind of a stereotype civil servant of always passing things up and down the line endlessly checking not only the facts but also whether the boss – and the boss’s boss and maybe the politician’s boss....you know, making sure everybody’s onside…And I think that one of the problems with the – certainly in government and to a certain extent with regulators – is sort of having somebody who can make that kind of authoritative statement …But I think that very often the problem is that they have people in all of the organisations who can’t really just act on 25% information, you know, they have to have 95%....you know, and 95% is incredibly difficult to get. (UK-IND11)

Furthermore, rather that working together, the message that was conveyed by our interviewees was that there is animosity and distrust between the politicians, the regulators and the industry. For example:

…the reason why politicians will get involved is because they will fear that not everybody in the food industry can be trusted, so that’s why regulation exists, is to prevent those that don’t play by the rules or don’t play fair from harming consumers and the general public. (UK-IND14)

The < name of regulatory body > is, sadly, a hollow shell of what it once. I mean food safety is all it does these days so, yes, you would expect the < name of regulatory body > to be a first port of call and to be doing something but they’re so emasculated these days you just think – it was a very, very clever move by the coalition government to not abolish it in the bonfire of the [inaudible] because I think there would have been an outcry and a massive campaign, not only by our campaigning sector but even some bits of the food industry wouldn’t have wanted to see it go. But what they wanted it to be was weaker and it now is. (UK-IND8)

UK-IND8, responsible for finance and management of industry, may be speaking to the recent (2010) changes in England whereby the main regulatory body is no longer responsible for food authenticity and composition, and are solely focused on food safety.

### Solutions for improving relationships

In order to address many of the potential areas of conflict mentioned above, participants voiced a variety of potential solutions to improve the relationships between regulators and industry. These included making regulation more flexible and realistic, encouraging information sharing and mutual education between regulators and industry, and generally encouraging more positive relationships through face to face interaction and frequent communication.

AU-REG respondents noted the need for a degree of flexibility in regulation, dependent on the industry involved. The degree of flexibility was identified as industry-specific, with changes in regulation likely affecting industries differently. This may be a potential solution to conflict, but one that needs to be approached with caution, taking into account the confusion and conflict that can result with inconsistency (i.e., differential treatment) and change:

I mean there’s a whole lot to food regulation now and there’s queries being raised by some whether the type of regulation, the outcomes based stuff that’s been put out there, is the best way to go or the prescriptive, or do you go like an island in the middle [allow some self-regulation]? The basis for the outcomes based is – so it gives industry the opportunity to be innovative and seek the outcome by a different means rather than traditional means and that’s fine. We’ve really only got so many businesses that have the capacity to do that, the majority – 99% – of businesses I’m sure, food businesses, haven’t got the capacity to be that innovative with how they comply with food regulation, so it’s – we’ll see over time whether that evolves. I’m sure it will but it’ll evolve to something else. There’s constant change which must be confusing for the industry. (AU-REG20)

As a solution to conflict between regulators and industry, AU and NZ regulation participants also discussed their role in ensuring that the regulations being put into place are realistic, requiring correspondence with and input from industry:

We need to make sure that it [regulation] is achievable by industry. It’s no good putting unrealistic requirements on industry, so we certainly sign off with industry that ‘here’s what we need to put in place and you tell us if you’ve got any concerns or if you think there’s any impracticalities with it and we’ll address those’. (AU-REG20)

Government and regulators can have a role helping the business to comply or knowing what they need to comply with rather than just setting it and hoping that they – or expecting that they do what they’re meant to do. (AU-REG19)

NZ regulator participants discussed the importance of communication to ensure that industry are able to understand, and more importantly, implement guidelines:

Part of having a good regulatory system is being good at voluntary uptake by industry and for us to have ways to assist them to do that by having some good guidance and information available. (NZ-REG6)

The importance of face-to-face interaction was also noted by state regulator AU-REG15:

I guess business owners and those sort of guys that are on the front foot, we have more of a collaborative approach when we work with them, so we’re definitely friendly, have a nice approach and talk with them and try and understand their business, their challenges. Being a smaller community we are quite visible to businesses so, you know, you pop into a shop and you buy an apple or you buy a drink or you get something and you have a chat with them and build a bit of a relationship that way. (AU-REG15)

AU industry also suggested a need for a relationship with regulators, and a means by which relationships can be developed between industry and regulators to facilitate information sharing. They viewed regulators as a resource:

I believe that it has to be win/win and I think when you’re building that relationship with them – I find with the health regulators, if you involve them in your business they will help you and anyone – I think once they know that you’re open to listening to them you build a rapport with them that then they’re willing to work with you but when you come in and see them as the enemy - oh my God the health inspector seen as an enemy - they’re not your enemy. They’re here and if you’re doing everything right - they’re your friend if you’re doing everything right and I think this is where some – I believe in the food industry some people say ‘oh they’re the enemy’. They are not the enemy they are the supposed industry experts and they’re sometimes more up to date than the mum and dad deli or the mum and dad restaurant. They’re more up to date with what’s going on… I think you have to use them as a resource rather than – I think they are a resource to you rather than anything else. (AU-IND9)

The AU industry participants recognised their need to partner with regulators, particularly in times of food safety incidents where government is seen as a credible source of information and arguably the representative of the food system:

In some cases our opinion is that government is going to have far more credibility than a commercial organization. We will always do our best but, you know, to have a government authority presenting the facts in some cases is far more credible than a commercial organization. (AU-IND6)

NZ industry participants spoke about the important role regulators play in the operation of industry. Discussing their role in advising on the recall of products, NZ-IND3 stated:

It was important that as an overarching regulator < name of regulating body > could provide good advice not only to the public but also to industry. (NZ-IND3)

The role of advisor is bidirectional, however. Regarding the lack of knowledge regulators have about the food industry, AU-REG3 (employed by a national food regulatory body) posed the solution of information sharing.

We have a good working relationship with the regulators. We give them information if they ask for it, and sometimes we give them information if they don’t ask for it but if we think it helps their position. I mean I think you need to understand that if the regulators are not close to the industry, if they don’t actually understand the industry itself, then they don’t – you know, they’re restricted in how effective they can be. (AU-REG3)

In the above quote AU-REG3 reflects the importance of a relationship between industry and regulators in the management of risk, with industry playing the role of educator in some circumstances. The regulators, in this case, need to work with industry to obtain the correct information about food safety issues in order to manage the risks.

In summary, AU and NZ regulator participants depicted greater understanding of the difficulties faced by industry in their compliance. This may be the result of their proximity to industry in the way regulation is enforced. For example, they discussed being able to meet face-to-face with industry, and to maintain continuity in regulatory representatives. As a result, they were able to suggest means by which barriers to communication and conflict could be overcome. As reflected by the lack of representation by UK in the above, UK interviewees did not discuss potential solutions for overcoming problems of distrust and lack of communication.

## Discussion

Consumers in industrialised countries increasingly demand foods that are safe and of high quality. This, alongside the interest of public health and an increasingly globalized food system, has led to the development of food safety standards [26]. This paper presents the views of individuals in food regulation (the regulators), and those in the food industry (the regulated), regarding their trust in and communication with one another within three countries: the UK, New Zealand, and Australia. Our primary aim was to provide recommendations, from representatives of industry and regulation, for facilitating the (re)building of trusting and communicative relationships between the two parties, with the end goal of increasing compliance with regulation and protecting public health. As an outsider, food safety standards may be viewed as a set of rules or best practices, enforced by one or more regulatory body. We may view industry then as compliant, or noncompliant. However, as we have shown, there are a variety of factors that shape the dynamic and complex relationships between regulators and industry, and the nature or extent of partial or (non)compliance. It is by speaking with these actors that we can identify barriers to compliance that are amenable to change. The following outlines key barriers and points of conflict identified in the data, with the primary aim being to propose solutions relevant to improving regulator-industry communication and trust. In doing so, we identify that while trust needs to occur at an interpersonal level between individual actors, trust at an institutional level also needs to occur. At times, participant comments were suggestive of a boarder distrust in either the systems of regulation or industry in general.

While previous research has identified that food safety regulation is trusted by the community [27], our data suggest this is not the case for individuals working within industry. Primarily, there is concern on the part of some UK and AU interviewees that regulations are based on larger political agendas or serve the interest of selected industry (rather than public health). Although these comments were not consistent across the findings, they are important nonetheless because they point to the need for greater transparency in the development and enforcement of food safety standards, and the need to develop institutional trust in regulation as a system – either at a local/state (e.g., department of health) or national level (e.g., FSA or FSANZ). Furthermore, these comments suggest a lack of, or breakdown in interpersonal trust between industry and regulators; a finding evident elsewhere in the data. Institutional distrust was also evident from the perspective of industry who at times suggested that regulation (rather than individual regulators) ‘holds’ industry down (e.g., red tape). Interestingly, regulator interviewees were aware of this perception and AU and NZ participants identified a need to communicate with industry about their actual intentions; ensuring public safety and safeguarding businesses. However, regulators were not always sympathetic to industry perceptions. There were concerns from regulators that individual industry representatives are at times purposely noncompliant with regulation, which reflected badly on industry generally. From the perspective of regulators, we suggest that interpersonal interactions with industry have tainted the perception of industry as a whole; a finding consistent with the theoretical literature on trust whereby it is argued that that although “the real repository of trust is in the abstract system, rather than the individuals who in specific contexts ‘represent’ it…it is the flesh and blood people (who are potentially fallible) who are its operators” [28] (p. 85) and who come to represent the system.

In several interviews from AU and NZ, success stories were noted, whereby participants discussed the importance of face-to-face communication between industry and regulators, establishing relationships at the onset of business development, and creating an environment where industry can enquire about food safety issues without fear of repercussion. We argue that these may be viewed as facilitators to the development of interpersonal trust between regulators and industry representatives, which based on our discussion above, may in turn have positive implications for institutional trust. Whilst there are undoubtedly structural and resource barriers to these solutions, they are nonetheless feasible in certain circumstances. For example, having one individual responsible for specific areas would allow for continuity in enforcement and the development of the interpersonal relationships and familiarity needed to foster trust [29].

Food safety is a complex issue and it is understandable that perspectives on how to manage food safety would differ between practical (industry) and technical (regulators) players. We argue that communication would facilitate a mutually beneficial understanding of these perspectives. For example, previous research has identified that in times of food safety incidents, it is important for public health professionals to work with the media in the construction of their reporting to ensure that information being disseminated to the public is accurate [30]. The need for information sharing (from industry to regulator) was identified in our interviews, whereby both parties indicated that some regulators lack knowledge about the food system which at times affects their judgement of how to handle food recalls, or their ability to relate to difficulties in meeting food standards. We suggest that a possible way forward is the inclusion in course curricula the study of food laws and standards for Environmental Health degrees, and for individuals in regulation who have not worked within the food system in any other capacity.

Most prominent in the findings from both parties were discussions around barriers to compliance. The perceived differential treatment, difficulty in understanding regulatory requirements, changing requirements and overregulation were concerns posed by industry, and recognised by NZ and AU regulators. Whilst a solution proposed by many to address overregulation was flexibility in enforcement, this may lead to further feelings of differential treatment and lead to confusion and individual interpretation of what/how standards should be met. Also problematic are the barriers to addressing changing requirements, as they are based on best-practice guidelines that are constantly being updated based on emerging research. However, based on the data from all three countries, we do recommend that an audit be conducted of what training, information and support is currently available to industry so that any recommendations made from the perceptions of respondents can be grounded in truth and tailored to complement existing resources. Furthermore, given the variety of industry (e.g., mom n’ pop stores, large corporations), individual communication with regulators could allow for adjustments to regulatory requirements on a case-by-case basis, though again there is room here to create confusion and conflict.

Also noted in the data is the importance of political climate and history in shaping how industry and regulators view one-another. Data from the UK were unique from AU and NZ interviews in that they were indicative of very poor relations between regulators and industry. In addition to conflict noted by AU and NZ participants, UK regulators perceived industry to be reluctant to work with them, while also noting that they did not see themselves as working ‘with’ industry but rather, their role was that of an enforcer. From an industry perspective, relationships with regulators lacked communication and trust. As UK-REG10 noted, this may be a problem rooted in history. We suspect that the findings are related to the restructuring of UK food standard governance over the past 15 years. In 2001, the Food Standards Agency (FAS) was developed in order to “put an end to the climate of confusion and suspicion which has resulted from the way food safety and standards issues have been handled in the past” [31] (p. 6). It was also created in response to the potential conflict of interest with the Ministry of Agriculture, Fisheries and Food (previously responsible for food safety) that arose from their dual role of protecting the interests of public health and agriculture and food industries [31]. The FSA was therefore purposefully developed so that it had institutional regulation of safety independence from producer interests, and also separated scientific advice from governmental departments, giving science a measure of institutional independence. These characteristics were deemed to be the features of an authority that helped to restore public trust in the UK food system [32]. However, in 2010, food authenticity and composition policy was transferred back to government departments. The UK National Audit Office (2013) state that this restructuring has led to confusion amongst food safety stakeholders: “local authorities…continue to be unclear on whom to contact, or get information from, in certain areas of food policy. They find that each department has a different approach and way of working which requires duplication of effort on their part.” (p. 7) [2]. Our data, consistent with the National Audit Office accounts, lead us to suggest that the delegation of responsibilities of the FSA be reconsidered. It is difficult to recommend further action given the complexity of bureaucratic processes. However, perhaps the use of industry advisory groups to inform the way forward for food standards would help develop or maintain trust. Furthermore, NZ and AU regulatory bodies may wish to look to historical blunders in any future plans for restructure.

## Conclusion

Building a strong interdependence between regulators and industry can balance power relationships, reduce misunderstandings, and ensure a reliable flow of information for both parties [14]. These are all crucial to the development and maintenance of trust and communicative relationships. Although the issues identified by interviewees differ, a common theme is the problematic lack of constructive communication. This points to the complexity of human relationships and the difficulty in streamlining processes that are dependent on context, political climate, individual behaviour, material resources, among other factors. Ideally, there should be greater communication between regulators and industry about why specific food standards are set (e.g., why it is important to have food stored at a particular temperature and to monitor the temperature). Furthermore, development of course curricula that includes increased workplace training for regulators on food practices would provide regulators with a greater understanding of the constraints placed on industry in meeting these regulatory requirements. In an evolving food safety climate, our paper offers insight into some of the barriers that shape noncompliance, and points to the tension and conflict identified between industry and regulators. As identified by participants, many of these conflicts and barriers may be easy to address with very few resources. Most central is the need for interpersonal communication from representatives of both parties. It clearly benefits public health to have transparent, open and communicative channels operating between food regulators and the food industry. We provide these data and insider suggestions as a means of overcoming conflict.

## Abbreviations

AU: Australia
IND: Industry
NZ: New Zealand
REG: Regulator
UK: United Kingdom

## Footnotes

1 Information regarding participant sample characteristics, as outlined in Table 2, have been included when reporting participant quotes if this information was deemed useful for the purpose of data interpretation.
